# ASAS-NANP symposium: mathematical modeling in animal nutrition: agent-based modeling for livestock systems: the mechanics of development and application

**DOI:** 10.1093/jas/skad321

**Published:** 2023-11-22

**Authors:** Karun Kaniyamattam, Luis O Tedeschi

**Affiliations:** Department of Animal Science, Texas A&M University, College Station, TX 77843-2471, USA; Department of Animal Science, Texas A&M University, College Station, TX 77843-2471, USA

**Keywords:** agent-based modeling, hybrid intelligent models, livestock systems

## Abstract

Over the last three decades, agent-based modeling/model (ABM) has been one of the most powerful and valuable simulation-based decision modeling techniques used to study the complex dynamic interactions between animals and their environment. ABM is a relatively new modeling technique in the animal research arena, with immense potential for routine decision-making in livestock systems. We describe ABM’s fundamental characteristics for developing intelligent modeling systems, exemplify its use for livestock production, and describe commonly used software for designing and developing ABM. After that, we discuss several aspects of the developmental mechanics of an ABM, including (1) how livestock researchers can conceptualize and design a model, (2) the main components of an ABM, (3) different statistical methods of analyzing the outputs, and (4) verification, validation, and replication of an ABM. Then, we perform an overall analysis of the utilities of ABM in different subsystems of the livestock systems ranging from epidemiological prediction to nutritional management to livestock market dynamics. Finally, we discuss the concept of hybrid intelligent models (i.e., merging real-time data streams with intelligent ABM), which have applications in artificial intelligence-based decision-making for precision livestock farming. ABM captures individual agents’ characteristics, interactions, and the emergent properties that arise from these interactions; thus, animal scientists can benefit from ABM in multiple ways, including understanding system-level outcomes, analyzing agent behaviors, exploring different scenarios, and evaluating policy interventions. Several platforms for building ABM exist (e.g., NetLogo, Repast J, and AnyLogic), but they have unique features making one more suitable for solving specific problems. The strengths of ABM can be combined with other modeling approaches, including artificial intelligence, allowing researchers to advance our understanding further and contribute to sustainable livestock management practices. There are many ways to develop and apply mathematical models in livestock production that might assist with sustainable development. However, users must be experienced when choosing the appropriate modeling technique and computer platform (i.e., modeling development tool) that will facilitate the adoption of mathematical models by certifying that the model is field-ready and versatile enough for untrained users.

## Teaser

Agent-based modeling for developing intelligent, sustainable livestock systems.

## Introduction

There are four significant paradigms for modeling agricultural systems: discrete events, dynamic systems, agent-based, and system dynamics, but the hybridization of more than one of these paradigms is often more effective for practical applications (Tedeschi, 2023). System dynamics or agent-based techniques have recently gained more advocates in developing modern computer models. All livestock systems can be construed as complex adaptive systems composed of heterogeneous and autonomous agents whose interactions drive system behavior (Nesheim et al., 2015). The agents in livestock systems can be heterogeneous regarding context, motivation, exposure to information, and scale of existence (Soriano et al., 2023). For example, agents in the system can be consumers, farmers, all the way down to animals, and bacteria. Another critical aspect of livestock systems is the ­existence of ­reinforcing and balancing feedback loops between system actors, which can give rise to unexpected emergent behavior (Diez Roux, 2011). Agent-based models (ABM; Macal and North, 2005), also known as the individual-based models, have been used by livestock researchers mainly to understand the social interaction of animals, to develop managerial solutions that can solve the environmental (Fust and Schlecht, 2018), economic (Kaniyamattam et al., 2018), and social (Huber et al., 2018) sustainability issues facing global livestock systems. A must-known difference between equation-based modeling and ABM is that the former is used to evaluate and predict, whereas the latter is used to emulate through specific attributes and attitudes (Tedeschi, 2023).

The onset of the fourth industrial revolution at the turn of the 21st century has witnessed exponential adoption of numerous cyber-physical systems (precision-livestock technologies, machine learning, artificial intelligence (AI), robotics, internet of things, and decision-support systems) by different livestock systems (Tedeschi et al., 2021). The resulting deluge of available data to the livestock systems should be efficiently utilized to achieve the multi-faceted sustainability challenges various livestock systems face. The one major disadvantage of applying data-driven methods (e.g., machine learning, deep learning) for livestock systems is that the generated data do not necessarily explain the relationships of variables in the system and the observed emergent patterns (Tedeschi et al., 2021). Agent-based models can be classified as conceptual or mechanistic, allowing them to simulate the observed behavior of a typical complex adaptive system such as a livestock system. It replicates the characteristics of individual agents in the system and agent-to-agent interactions, thereby simulating the observed emergent behavior of a livestock system. Hence the objective of our review paper is to provide an overview of the potential of ABM, discuss the processes involved in the development dynamics of ABM, and introduce livestock systems decision modelers to state-of-the-art ABM research being conducted in the field of animal science and related areas. A case for the need and utility of hybridizing ABM with other AI-based techniques (e.g., machine learning and computer vision) is also discussed for developing hybrid intelligent decision-making models to achieve sustainable livestock systems (Tedeschi 2023).

## Definitions and Characteristics

ABM is a computational model used to mimic and study real-world complex systems using mathematical modeling (Bonabeau, 2002), which encodes the behavior of individual agents in a population and their interactions. Understanding complex systems and their emergent patterns using one’s senses is difficult for most people, even for field experts. The difficulty to perceive and process emergent patterns (Wilensky and Rand, 2015) could be broadly classified into two reasons: (1) limited integrative understanding, where one knows about the individual behavior but fails to comprehend the aggregate behavior of the population, or (2) limited differential behavior where the aggregate population behavior is easily perceived but the behavior of elements which make up the pattern is difficult to perceive. ABM is a suitable computational representation that can help elucidate the integrative and differential behavior of complex systems under study. Most ABM are designed to capture the emergent properties and dynamics arising from the interactions between agents and their environment. ABM’s key features and components that make it different from other modeling methodologies are the agents, interactions among agents, environment, rules and behaviors of the agents, and the simulation process.

### Critical features of agent-based models

#### Agents

**Figure 1.**
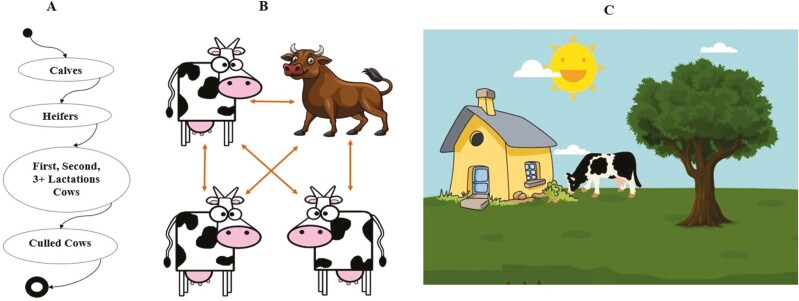
A general framework used for designing agent-based models is adapted for a typical cow herd modeling scenario. (A) Illustration of the temporal behavior of representative cow agents which were initiated as calves in the herd, which ages to become heifers, which when pregnant progress through first, second, and third lactations, before being culled from the herd. (B) depicts the scenario where agents (cow/bull in this depiction) interact with each other. (C) shows a scenario where a cow can interact with their environment. Adapted from Metcalf (2007).

Agents are the fundamental entities within an ABM (Figure 1). They can represent various entities such as individuals, organizations, animals, or other relevant actors in the modeled system. Each agent has its attributes, memory, autonomy, behavioral rules, and decision-making protocols (Macal and North, 2005) that result in controlling their interactions with other agents and the environment. Agents can change their characteristics and, possibly, rules as time goes by. For example, calves can become heifers, first lactation, second lactation + cows as time progresses to replicate the physiological processes happening in a biological agent (Figure 1).

#### Interactions

**Figure 2.**
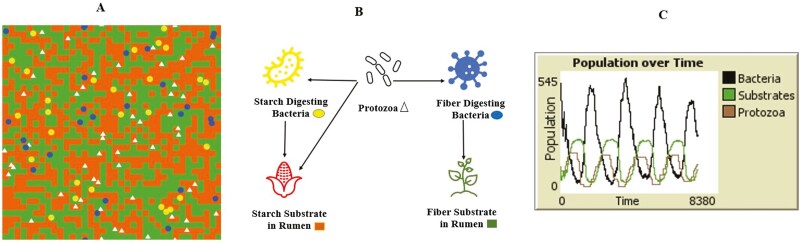
A simplified pictorial depiction of an agent-based model of the rumen fermentation process. (A) has the spatial (within the boundaries of the rumen) visualization of the agents: red represents starch and green represents fiber in the rumen. (B) has more focused visualization, showing the interaction between agents (predators) and the environment (prey) in the model. The fiber-digesting bacteria (yellow circles), starch-digesting bacteria (blue circles), and protozoa (white triangles) are the three different interacting agents (i.e., predators) in the model. Fiber-digesting bacteria consume the fiber in the rumen. Starch-digesting bacteria and protozoa digest starch. Protozoa also consume both types of bacteria. (C) The emergent behavior is the population dynamics in the model, inspired by Lotka-Volterra differential equations. The number of predators (protozoa, fiber, and starch-digesting bacteria), number of prey (fiber, starch, fiber, and starch-digesting bacteria), and the growth and death rate of prey population along with the daily substrate replenishment into rumen affect the constant cyclical model behavior. Underlying behavior adapted from Wilensky and Rand (2015a).

ABM focuses on capturing the interactions and relationships between agents. Figure 1 shows the cow-to-cow interaction, and Figure 2 depicts bacterial and protozoal interactions. Agents can communicate, exchange information, collaborate, compete (bacteria and protozoa compete for starch substrate in Figure 2), or influence each other’s behaviors and decision-making processes (protozoa predate bacteria) (Tedeschi and Fox, 2020; Osorio-Doblado et al., 2023). These interactions can be direct, indirect, or mediated through the environment. For instance, in the rumen fermentation model shown in Figure 2, an increase in the protozoa preying on bacteria leads to a decrease in bacterial population over time. When the bacterial population decreases, the protozoal population decreases as there is not enough substrate to feed on. As the bacterial population increases again in the rumen in the presence of starch and fiber substrates, the pattern repeats, maintaining a cyclical process of substrate digestion and growth in ruminants. Protozoa, nonetheless, not only predate on bacteria, but also meet their energy and protein needs from the fermentation of simple and complex carbohydrates (e.g., sugars, fructose, cellulose, and starch). Thus, adding this third relationship (protozoa engulfing carbohydrates) drastically increases the system’s complexity, as bacteria are not the only prey in the environment. Figure 2 illustrates multiple trophic layers of interaction between agents (substrates, bacteria, and protozoa) using the ABM approach.

#### Simulation environment

Within an ABM structure, the modeling and simulation (a better term is emulation) environment refers to the context or setting in which the agents operate. It includes the physical space, resources, constraints, and other external factors influencing agent behavior. The environment can change dynamically, and agents can respond and adapt to these changes (e.g., shade, sun, grass availability in Figure 1) that affect cow behavior. The availability of fiber and starch in the rumen affects the population dynamics of rumen bacteria and protozoa (Figure 2). Environmental variations in an ABM can include weather, temperature conditions, seasonality (Namany et al., 2020), or natural disturbances. The environment in ABM can be represented using different unique structures, such as grids (Lytinen and Railsback, 2012), networks, or continuous spaces. The environment provides resources that agents require for their survival, growth, or reproduction (e.g., fiber and starch substrates for bacteria and protozoa, as shown in Figure 2).

#### Rules and behaviors

As discussed above, agents in an ABM follow specific rules and behaviors that guide their actions and decision-making, and it is part of their expected interaction. These rules can be simple or complex, and they define how agents perceive, process information, respond to their environment, and interact with other agents. Agent behaviors can include learning, adaptation, memory, and decision-making based on heuristics (Sun et al., 2016) or explicit algorithms. For instance, in the case shown in Figure 1, an agent initiated as a calf is shown to mature into a heifer capable of being pregnant and bearing another calf. As time progresses, cows can age and become culled from the herd at any point in time, depending on the farm’s economic or managerial decision rules.

### Emergent properties of the simulation process

A desired goal for an ABM is to capture the emergent properties (Macal and North, 2005) that arise from the interactions of individual agents. These properties are system-level patterns, behaviors, or phenomena that cannot be directly attributed to the actions of any single agent. Random individual behavior can result in consistent patterns of population behavior known as emergent behavior. These complex population-level patterns can self-organize without any leader orchestrating this behavior. ABM helps understand how macro-level phenomena, such as collective behaviors or global outcomes, emerge from the micro-level interactions of agents. In the rumen fermentation model shown in Figure 2, the only goal of fiber-digesting bacteria (random individual behavior) is to digest the fiber available in the rumen. If there is continuous availability of the substrate (i.e., fiber), the population of fiber-digesting bacteria will increase exponentially, assuming a conducive environment (e.g., absence of bacteriocin and normal volatile fatty acid concentration) that does not limit bacteria growth. However, protozoal predation and cyclical availability of fiber in the rumen ensure that the fiber-consuming bacteria population oscillates within a high and low interval range, aiding in the optimal digestion cycle conducive for cow growth but yielding a dynamic equilibrium among the agents. The emergent property of ABM is used to mimic a bottom-up approach in which the overall behavior stems from the interactions among agents, whereas system dynamics is used on top-down approaches in which the behavior dictates the model structure (Tedeschi, 2023). The model depicted in Figure 2 is a simplification of the in vivo fermentation process of a cow, designed to illustrate ABM model development mechanics. The fiber digestion in the rumen is a multispecies-multifactorial driven complex process, with innumerous levels of substrate-microbe interactions, parts of which are yet to be understood by ruminant nutritionists (Osorio-Doblado et al., 2023).

ABM are used for simulation, allowing researchers to observe the system’s dynamic behavior over time. Figure 2C shows the rumen’s dynamic behavior of bacteria, protozoa, and substrates. By simulating the actions and interactions of agents, an ABM generates outputs that represent the evolution of the system, enabling the study of different scenarios, interventions, or policy changes. ABM provides a flexible and versatile approach to modeling complex systems, including social, biological, ecological, and economic systems. They offer a bottom-up perspective, focusing on individual agents’ behaviors and interactions, allowing for a more realistic representation of real-world scenarios. ABM are used in various fields, including social sciences, economics, ecology, epidemiology, transportation planning (Kagho et al., 2020), and many others, to gain insights into the dynamics and behavior of complex systems.

## Agent-Based Models for Livestock Systems

Complex adaptive systems are characterized by multiple interconnected components or agents that exhibit adaptive behaviors in response to environmental changes or interactions with other agents (Holland, 1992). With their inherent complexity and adaptive behaviors of the various system components, livestock systems are ideal examples of complex adaptive systems (Gross et al., 2006). Some key characteristics of livestock systems that align with the concept of complex adaptive systems and thereby favor using ABM to study them include multiple interacting agents, nonlinear dynamics, adaptation and learning, emergent properties, uncertainty and resilience, and the interdisciplinary focus.

### Multiple interacting agents

Livestock systems involve various agents, such as animals, farmers, consumers, and other stakeholders. ABM allows researchers to represent the diversity and heterogeneity of agents within a livestock system, and it also accounts for variations in agent characteristics, behaviors, and decision-making processes. These agents interact with each other, making decisions and influencing the whole system’s behavior. The actions and behaviors of one agent can have ripple effects on others, leading to emergent behaviors and system-level patterns. This individual-level approach enables a more realistic representation of the system under study and its dynamics, thus, creating the expected bottom-up behavior.

### Nonlinear dynamics

Livestock systems often exhibit nonlinear relationships and feedback loops (Groves et al., 2022). Minor changes or disturbances within the system can lead to disproportionate or unexpected consequences. This nonlinearity is driven by the interactions and feedback between agents and their environment, contributing to the complex dynamics of the system. ABM can capture complex dynamics such as feedback loops, self-organization, and collective behaviors within livestock systems by simulating agents’ behavior over time, providing insights into how the system responds to various interventions or changes.

### Adaptation and learning

Livestock systems also exhibit adaptive behaviors, where agents adjust their strategies and behaviors in response to changing conditions. Animals adapt to their environment, farmers adjust their management practices (Yang et al., 2021), and consumers adapt their preferences and demands. This adaptability allows livestock systems to respond to internal and external pressures and optimize their performance under varying circumstances. ABM can be used to assess the effects of different management practices, interventions, or policy decisions on livestock systems’ performance, sustainability, and resilience. Researchers can simulate various scenarios and evaluate the outcomes, allowing for informed decision-making and policy formulation.

### Emergent properties

As expected, livestock systems can display emergent properties that arise from the interactions and behaviors of individual agents. These emergent properties may include self-organization (Noe and Alrøe, 2003), system-level patterns, and collective behaviors that cannot be attributed solely to the actions of individual agents. The overall behavior of the livestock system emerges from the complex interactions and feedback loops between its components. If one simulates the rumen fermentation model shown in Figure 2B and 2C, one can appreciate the cyclical population dynamics of bacteria, protozoa, and substrates over time in the rumen environment. When substrates are abundant, the bacterial population prospers, which has a reverberating effect of increasing the protozoal population, and reducing the bacterial population. Hence the interaction of the bacterial population with substrate availability impacts both the bacterial and protozoal population densities at different rates. These are examples of emergent phenomena that arise from the simple rules of eat, reproduce, and die encoded into the NetLogo program code used to develop the model (Supplementary Appendix 1).

### Uncertainty and resilience

Like many agricultural activities, livestock systems operate in uncertain environments, influenced by climate variability, disease outbreaks, and market fluctuations. These systems demonstrate resilience by adapting to disturbances, recovering from shocks, and maintaining their functionality over time. ABM can help researchers anticipate and understand the potential risks and uncertainties (Baustert and Benetto, 2017) associated with livestock systems. By incorporating stochastic elements and simulating a range of scenarios, ABM allows for assessing system vulnerabilities, disease spread, resource limitations, and other factors impacting livestock production and sustainability.

The intrinsic characteristics of livestock production systems of being complex and multidimensional, involving aspects of animal science, economics, ecology, and social sciences, make them suitable for interdisciplinary research. ABM provides a platform for integrating knowledge and expertise from various disciplines, fostering multidisciplinary collaborations (Axelrod, 2006) with a holistic approach to assist with livestock systems research.

## Platforms for Designing and Building Agent-Based Models

Given the rising adoption of ABM by different science disciplines, several modeling communities throughout the last few decades have built several software platforms for designing and building ABM (Gilbert and Bankes, 2002). Even though all platforms provide tools and frameworks for creating, analyzing, and visualizing ABM, they all have distinctive characteristics depending on the programing language used, the model development effort required, and the model’s scalability. Abar et al. (2017) have done a comprehensive comparative literature survey of 85 different state-of-the-art software platforms globally available for ABM development. The Network for Computational Modeling in Social and Ecological Sciences (CoMSES Net; Janssen et al., 2008) is an open-source community of ABM researchers that hosts hundreds of peer-reviewed ABM, enabling the re-use of ABM codes by peer modelers. These software platforms differ in programming languages, features, ease of use, and target applications. Researchers can choose the platform that best suits their specific modeling needs, programming skills, and complexity required for their ABM. Below are some commonly used software platforms for ABM development with distinct characteristics.

### NetLogo

NetLogo (Wilensky, 1999) is an open-source, user-friendly, and widely used platform specifically designed for building ABM, developed by Uri Wilensky of Northwestern University, Illinois, USA. It provides an intuitive graphical interface and a language adapted based on the original Logo. It uses architectural agents consisting of turtles, patches, links, and the observer for ABM development. NetLogo supports creating models with multiple agent types, customizable behaviors, and interactive visualizations (see Figure 2).

### Repast J

Repast (Recursive Porous Agent Simulation Toolkit) J is a Java-based open-source software platform for building ABM, developed by Argonne National Laboratory (North et al., 2013). Repast J’s framework (North et al., 2005) allows researchers to design and implement complex models with multiple agents, agent interactions, and environmental ­components. Repast J provides libraries, tools, and visualization capabilities (North et al., 2007) for ABM development. The object-oriented design of Repast J ensures that both the library and user models are flexible and are typically used to build highly scalable ABM.

### AnyLogic

AnyLogic (Borshchev, 2013) is a commonly used powerful multi-method simulation software that supports the development of various model paradigms, including ABM. It offers a graphical modeling environment and supports different ABM approaches, such as discrete-event, system dynamics, and ABM. AnyLogic allows for integrating different modeling paradigms (Borshchev et al., 2002) within a single model and requires a proprietary license to build high-end models.

### Swarm

Swarm is an open-source platform for general-purpose ABM developed by the Swarm development group (Minar et al., 1996). It provides a flexible and extensible environment for creating agent-based models in Java and Objective-C. Swarm focuses on individual-based modeling and supports the simulation of large-scale agent populations and complex agent behaviors.

### Mason:

Mason is a Java-based ABM toolkit that focuses on high-performance computing, developed by George Mason University, Virginia, USA (Luke et al., 2005). It provides a lightweight and efficient framework for developing large-scale ABM. Mason emphasizes speed and scalability and offers features such as grid computing and parallel execution.

### Generic architecture for multi-agent systems

Generic architecture for multi-agent systems (GAMA) is an open-source platform (Luke et al., 2005) for building agent-based models. It supports modeling and simulating various systems, including social, ecological, and economic systems. GAMA is written using Gama Modeling Language, providing a visual modeling environment and a flexible scripting language for agent behaviors.

### Mesa:

Mesa (Masad and Kazil, 2015), developed using Python-3, has a similar implementation design as NetLogo, Repast J, or Mason. It enables modelers to visualize simulations in browser-based interfaces. Modelers can also analyze simulation results using Python’s data analysis tools.

## Conceptualizing Agent-Based Models for Livestock Production Systems

**Figure 3.**
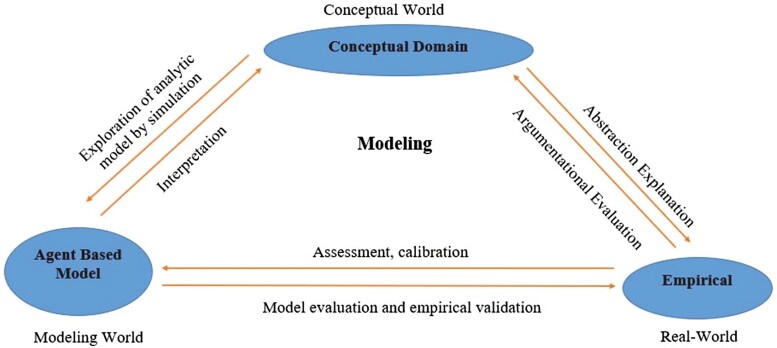
A conceptual depiction of the interconnectedness of the real-world system, conceptual and agent-based modeling domain which illustrates the iterative nature of agent-based model development. Adapted from Tedeschi (2019).

**Figure 4.**
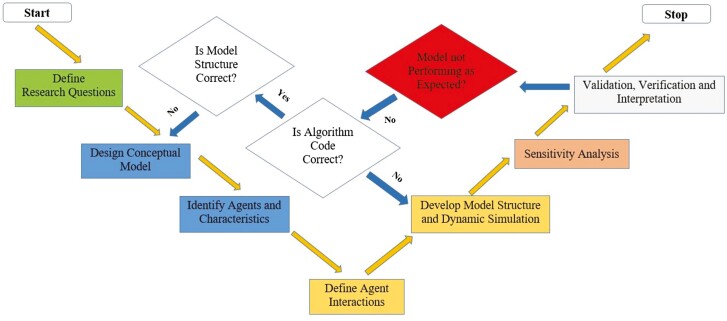
A flow diagram illustrating the sequential process, which livestock system researchers can follow for agent-based modeling to develop a valid and verifiable model of a real system. The arrows in the center indicate that all of these processes are iterative, if there is error in one of the processes. Color schematics and process flow adapted from Tedeschi (2023).

In the case of livestock systems, the agents in the model could be animals, herds, farmers, consumers, or other relevant actors in the system (Fernandez-Mena et al., 2020), depending on the research questions that need to be answered by the model. A team of livestock systems researchers could follow the following key steps to conceptualize and design (Figures 3 and 4) an ABM customizable for their needs. By following these steps, a livestock systems researcher can promptly develop a useful tool for exploring the dynamics of livestock systems and informing decision-making for sustainable farming (Gutiérrez et al., 2017).

### Establishing the research question

The researchers should clearly define the research (Figure 4) question they want to address with the ABM. A representative research question can be constructed as follows: investigate the differential managemental strategies impacting pasture-based beef cattle production systems. For example, Addis et al. (2022) developed an ABM representing an integrated pasture-based beef cattle finishing system in New Zealand. Their research objective was to improve beef production from dairy breeds of cattle. Yet another recent ABM developed by Yang et al. (2019) investigated a key beef operations management question related to cattle transportation under unforeseen disturbances to the system. Thus, clearly defining the research question for the modeling team is a crucial requirement before venturing out with the model development per se.

### Designing conceptual models

According to Wilensky and Rand (2015), there are two major modeling categories: phenomena-based and exploratory. In phenomena-based modeling, modelers try to replicate a reference pattern from the system behavior. For example, the famous housing segregation model in cities by Schelling (1971) reproduces the housing pattern of a specific town based on the city populations’ propensity to live among people from different ethnic groups. ABM was developed for the second category of modeling, i.e., exploratory modeling creates a set of agents, defines their behavior, and explores the patterns that emerge, which is more of a bottom-up approach commonly followed by ABM. One significant other example is the Game of Life model (Adamatzky, 2010) developed by John Norton Conway in 1970. Once an initial pattern of agents is defined, the replication patterns of agents in the ­environment are self-evolving. Hence depending on the research questions and type of patterns researchers are interested in, an ABM approach will involve designing a conceptual model (Figure 4) and its expected behavior. Ideally, this conceptual model and its system boundaries should be decided upon before embarking on making the model dynamic.

### Identifying the agents and their characteristics

The researchers should identify the agents included in the model and their characteristics (Figure 3). For example, in a pasture-based beef management model by Addis et al. (2022), the multi-tier agents could be cows, farmers, and grass. The cows could have attributes such as breed, age, weight, and grazing preferences, while farmers could have characteristics such as management practices and financial constraints. Depending on the modelers’ viewpoint, almost any component in a real system can be an agent. Sufficient thought should go into the granularity of the model to ensure that the model behavior is manageable. The required characteristics of the agents should be decided so that when agents dynamically interact among themselves and with their environment, the expected system behavior can be simulated. For example, in the rumen fermentation model (Figure 2), bacteria and protozoa ferment substrates, expend energy, reproduce, and die, thereby affecting the population dynamics of two species that co-exist.

### Defining agent interactions

The researchers should define how the agents interact (Figure 3) with each other and their environment. For example, Ojeda-Rojas et al. (2021a), for their ABM, which compared the different reproductive strategies in cow-calf operations, modeled the interaction between dry cows, heifers, male, and female calves, milking cows, and bulls (other agents in the model) to simulate the reproductive performance of typical Brazilian beef cattle operations. In the bacteria-protozoa model (Figure 2), bacteria can consume rumen substrates, while protozoa can consume both rumen substrate and bacteria.

### Developing the model structure and dynamic simulation of the agent-based model

The researchers should develop the structure of the model, including the equations or algorithms that represent the interactions between agents. The model could be programmed using software such as NetLogo, Repast J, or AnyLogic, as discussed above. Once the basic components of ABM are designed and modeled, one should design the typical time step in the model. The specific behaviors of the agents in the model, the sequence in which different behaviors are exhibited, and the probability of different events happening in the agent’s lifecycle, as well as in the environment. The agents need a set of parameters that control their behavior along the simulation time window. In the rumen fermentation model, examples of parameters would be the number of bacteria and protozoa, movement cost, energy gain from substrates, feed replenishment rate, and so forth.

### Conducting sensitivity analysis

The researchers should conduct different sensitivity analysis techniques (Thiele et al., 2014) to assess the robustness of the ABM by changing the parameters and assumptions. The most straightforward approach is doing one-factor-at-a-time simulation and manually ensuring that the system’s key performance measures are behaving as expected. The design of experiment principles of changing different levels of parameters in the model (Ten Broeke et al., 2016), is yet another approach when a model has multiple parameter levels. Some ABM programming platforms have the inbuilt capability to do sensitivity analysis. For instance, NetLogo has a feature called Behavior space (Thiele et al., 2014), which can automatically run multiple batches of in silico experiments, each experiment with multiple combinations of parameter levels.

### Validating, verifying, and replicating the mathematical model

The researchers should validate and verify the model (Figure 4) by comparing its output to real-world data, for a model to be helpful in answering real-work questions (Tedeschi, 2006; Windrum et al., 2007). This process ensures that the model is accurate, reliable, and carries out its intended function. There should be constant to and from communication between the domain expert and the model builders to ensure that the model is valid and robust, as in silico modeling of a real-work complex system is an iterative process. While validations and verification words sound synonymous, there are inherent differences in the actual processes carried out (Tedeschi, 2006; Ormerod and Rosewell, 2009). Model validation corresponds to determining whether the implemented model explains some phenomena in the real world. In contrast, model verification determines whether the developed model corresponds to the target conceptual model (Tedeschi, 2006). To validate the ABM, researchers should decide on the critical performance measures that they collect from the simulation. ABM data could be examined and analyzed using different ways. Researchers should decide on the analysis methods before building the ABM, to enable data outputs that are appropriate to the analysis. As stochasticity is an inherent nature of agents, (because of routine sampling from distributions, for agent’s behavior parameters) agents are heterogenous in an ABM, and hence most of the key performance measures from model runs will be different. Running multiple iterations of the dynamic model will mitigate the effects of heterogeneity in results. The mean and standard deviation of simulated behavior measures are often compared to real-world data to validate ABM. The process of ensuring that an implemented model corresponds to a conceptual model (verification) as well as to the data outputs exhibited by the real-world systems (validation), is paramount to ensure the confidence (Wilensky and Rand, 2007) of the research community in the correctness and explanatory power of both the conceptual and implemented models. ABM should be replicable across multiple platforms and programming languages to augment confidence among the scientific community in decisions predicted by ABM. Despite the known benefits, replication of ABM occurs very infrequently (Wilensky and Rand, 2007). The replication process supports both the verification and validation process, as the replicated model ensures that the ABM developed using a different platform corresponds to both the real-world scenario and conceptual model construed by modelers (Tedeschi, 2019).

### Interpret the results

The researcher should interpret the model’s results in the context of the research question. The model generated insights into the system-level behavior, especially the system performance under different scenarios or management practices, as well as agent-level behaviors that should be analyzed critically. Patterns and trends from the system should be compared across various strategies, interventions, or policy changes. This kind of analysis can help identify influential agents, bottlenecks, or critical points (Wilensky and Rand, 2015) within the livestock system. Overall, interpreting the results from an ABM simulation requires a deep understanding of the system being modeled and a rigorous approach to model development and validation, which involves multiple iterative steps ranging from defining research questions, to modeling, to validation (Figure 4).

## Extant Agent-Based Models for Livestock Systems

Over the course of the last two decades, numerous ABM have been developed in various areas of livestock systems research, including epidemiological models, grazing, and foraging behavior, livestock market dynamics and production systems, and land use.

### Disease spread and control

ABM has been used for quite some time to simulate the spread of infectious diseases within livestock populations and evaluate control strategies. These models consider individual animals as agents and simulate their movements, interactions, and disease transmission dynamics. ABM are quite often used to simulate and assess the spatiotemporal spread of livestock diseases (foot-and-mouth disease (Roche et al., 2014), bovine respiratory disease (Thompson, 2021), and African swine fever disease (Lange and Tulke, 2017)), as discussed by Bradhurst et al. (2016). They devised a technique for researchers with access to modest hardware platforms to simulate the spread of illness on a large scale. Wiltshire (2018) built a regional ABM for US-based hog production networks to assess the potential for catastrophic disease outbreaks. They found that connectivity patterns of contact networks often predict epidemic spreading dynamics. ABM can help evaluate the effectiveness of vaccination programs, biosecurity measures, and other interventions in mitigating disease spread. Thompson et al. (2021) investigated the emerging threats from antimicrobial resistance to the beef industry’s sustainability.

### Grazing and foraging behavior

ABM have been employed to study livestock’s foraging (Dumont and Hill, 2001) and grazing (Yu et al., 2019) behavior in relation to resource availability and environmental conditions. These models simulate individual animals’ decision-making processes, considering factors such as remote sensing information about grasslands, forage quality, pasture accessibility, grassland degradation in response to grazing management (Yu et al., 2019), and social interactions. ABM can help understand grazing patterns, resource utilization, and their implications for livestock productivity and environmental sustainability.

### Livestock market dynamics

The dynamics of the livestock market have been modeled using ABM (Schreinemachers and Berger, 2011) to analyze the impact of various factors on market outcomes. These models represent individual farmers, traders, and consumers as agents and simulate their decision-making processes regarding buying, selling, and pricing livestock. ABM can help assess market behavior, price volatility, and the effects of policy interventions (Marvuglia, et al., 2022) on livestock markets. Yang et al. (2019) used ABM approach to simulate beef cattle production and transportation in Southwest Kansas.

### Livestock production systems

Few ABM have been used to simulate livestock production systems and evaluate management strategies for improving productivity and sustainability (Bayram et al., 2023). These models consider individual animals, farmers, and environmental factors as agents. These ABM capture animal interactions, feeding and growth processes, farm management practices, and environmental conditions. They can help optimize feed allocation, assess the environmental impacts of different management practices, and explore tradeoffs (Marvuglia et al., 2022) between productivity and sustainability. Ojeda-Rojas et al. (2022) compared cow-calf operations’ performance under different reproductive strategies using an ABM.

### Land use and environmental impacts

The interaction between livestock systems and land use change have been assessed with ABM (Dressler et al., 2019), as well as environmental impacts. These models represent livestock production, land management, and environmental processes. ABM can help analyze the effects of land use decisions, such as deforestation (Müller-Hansen et al., 2019) or conversion of pasture to cropland, on livestock production, greenhouse gas emissions, biodiversity, and other environmental indicators.

These examples demonstrate the diverse applications of ABM in livestock systems research, ranging from disease dynamics and market analysis to environmental sustainability and land management. ABM provides a valuable tool for studying the complex interactions and dynamics within livestock systems, enabling researchers to investigate various aspects of livestock production, management, and policy.

## Hybrid Intelligent Modeling to Augment ABM Capability

Hybrid Intelligent Modeling (HIM) refers to integrating multiple modeling approaches, including ABM, with other computational techniques like AI (Zhang et al., 2021). In the context of livestock systems research, HIM using ABM enhances the model’s capabilities, realism (Tedeschi et al., 2021), and predictive power. As data-driven models, which utilize machine learning and AI, as well as ABM have their own merits and demerits, the efficient synthesis of these methods is often the solution to explaining complex system behaviors. The potential of this kind of (Tedeschi, 2023) synthesized model, often referred to as a hybrid intelligent mechanistic model (HiMM) or intelligent agent-based model (iABM), is still underutilized by livestock systems for attaining its sustainable development goals (Jacobs et al., 2022; Tedeschi, 2023). The HIM could be efficiently used to accurately predict greenhouse gas (GHG) footprint from enteric and manure-based emissions of ruminants. Developing a HIM for cattle emissions could potentially entail a three-step integration of (1) mechanistic models, which simulate the digestive process, methane production, and manure management practices, (2) machine learning models trained on historic data on GHG emissions under different diet composition, feeding practices, and environmental conditions, and (3) integration of real-time data from GHG sensors and precision technology monitoring animal behavior, with rule-based expert systems (i.e., ABM parameterized with machine learning based models) about enteric and manure emissions. Below are some key aspects and benefits of using HIM with ABM in livestock systems research.

### Integration of multiple modeling paradigms

The HIM allows for the integration of different modeling paradigms, such as ABM, system dynamics, optimization, or machine learning, within a single framework. This integration enables a more comprehensive representation of the livestock system by capturing different aspects, behaviors, and feedback mechanisms, thereby ensuring real-time decision-making.

### Agent behavior and decision-making processes

The ABM focuses on modeling individual agents and their behaviors. The HIM can replicate the real-time behavior of real-world agents, and incorporate cognitive learning by simulated agents, allowing them to make more informed and realistic decisions based on available information, learning (Garcia et al., 2020), or optimization algorithms.

### Data-driven modeling

The HIM can leverage machine learning techniques to inform agent behavior or model calibration. By integrating data-driven models, such as predictive analytics or data mining, with ABM, researchers can improve the accuracy and predictive capabilities of the model by incorporating observed patterns or historical data.

### Adaptive and learning agents

The HIM enables the modeling of adaptive and learning agents within ABM. Agents can dynamically adjust their behaviors, strategies, or decision-making processes based on feedback, past experiences, or changing environmental conditions. This allows for a more realistic representation of livestock systems where agents can adapt to changing circumstances.

### Enhanced prediction and policy evaluation

The HIM can improve the model’s predictive power and support policy evaluation. By combining ABM with optimization techniques or machine learning algorithms, researchers can identify optimal management strategies, evaluate policy interventions, or predict the outcomes of different scenarios with greater accuracy.

### Robustness and sensitivity analysis

The HIM allows for robustness and sensitivity analysis by integrating different modeling approaches. Researchers can assess the stability and sensitivity of the model outputs by exploring the effects of parameter variations, uncertainty, or alternative modeling assumptions across different modeling paradigms.

### Visualization and communication

The HIM provides opportunities for advanced visualization techniques to effectively communicate the model’s outputs and insights. Visualizations can help stakeholders and decision-makers (Bansal et al., 2016) better understand complex livestock systems and the implications of different scenarios or policy choices.

By combining the strengths of ABM with other computational techniques and AI methods, HIM in livestock systems, research can lead to more comprehensive, realistic, and powerful models. It allows for a deeper understanding of livestock systems’ dynamics, behaviors, and interactions and supports decision-making processes for sustainable and efficient livestock management.

## Conclusions

The ABM paradigm offers researchers a powerful tool to understand and analyze the complex dynamics of livestock systems. Agent-based models capture the characteristics of individual agents, their interactions, and the emergent properties that arise from these interactions. Livestock systems researchers can benefit from ABM in various ways, including understanding system-level outcomes, analyzing agent behaviors, exploring different scenarios, and evaluating policy interventions. To design and build ABM, researchers can utilize software platforms developed explicitly for ABM development, such as NetLogo, Repast J, AnyLogic, Swarm, Mason, or GAMA. Livestock systems researchers should carefully conceptualize and design ABM, considering the research objectives, system components, agent behaviors, and available data. Notable ABM developed by livestock systems researchers can provide valuable insights and serve as references for future model development. Moreover, augmenting ABM with HIM techniques, such as incorporating machine learning or optimization algorithms, can enhance their capabilities, predictive power, and realism. Hence, ABM offers a versatile and promising approach to studying livestock systems, providing valuable insights into their dynamics, behaviors, and sustainability. By combining the strengths of ABM with other modeling approaches, researchers can further advance our understanding and contribute to sustainable livestock management practices.

## Supplementary Material

skad321_suppl_Supplementary_Appendixs_1

## Abbreviations

ABM: agent-based model
AI: artificial intelligence
GAMA: generic architecture for multi-agent systems
HIM: hybrid intelligent modeling
iABM: intelligent ABM
IBM: individual-based model

## References

[CIT0001] Abar, S., G. K. Theodoropoulos, P. Lemarinier, and G. M. O’Hare. 2017. Agent-based modelling and simulation tools: a review of the state-of-the-art software. Comput. Sci. Rev. 24:13–33. doi: 10.1016/j.cosrev.2017.03.001

[CIT0002] Adamatzky, A. 2010. Game of life cellular automata. London: Springer. ISBN: 978-1-84996-216-2

[CIT0003] Addis, A. H., H. T. Blair, P. R. Kenyon, S. T. Morris, N. M. Schreurs, and D. J. Garrick. 2022. Agent-based modeling to improve beef production from dairy cattle: model description and evaluation. Agriculture 12:1615. doi: 10.3390/agriculture12101615

[CIT0004] Axelrod, R. 2006. Agent-based modeling as a bridge between disciplines. Handb. Comput. Econ. 2:1565–1584. doi:10.1016/S1574-0021(05)02033-2.

[CIT0005] Bansal, S., G. Chowell, L. Simonsen, A. Vespignani, and C. Viboud. 2016. Big data for infectious disease surveillance and modeling. J. Infect. Dis. 214:S375–S379. doi: 10.1093/infdis/jiw400 28830113 PMC5181547

[CIT0006] Baustert, P., and E. Benetto. 2017. Uncertainty analysis in agent-based modelling and consequential life cycle assessment coupled models: a critical review. J. Clean Prod. 156:378–394. doi: 10.1016/j.jclepro.2017.03.193

[CIT0007] Bayram, A., A. Marvuglia, T. N. Gutierrez, J. Weis, G. Conter, and S. Zimmer. 2023. Sustainable farming strategies for mixed crop-livestock farms in Luxembourg simulated with a hybrid agent-based and life-cycle assessment model. J. Clean Prod. 386:135759. doi: 10.1016/j.jclepro.2022.135759

[CIT0008] Bonabeau, E. 2002. Agent-based modeling: methods and techniques for simulating human systems. Proc. Natl. Acad. Sci. U.S.A. 99:7280–7287. doi: 10.1073/pnas.082080899 12011407 PMC128598

[CIT0069] Borshchev, A . 2013. The Big Book of Simulation Modeling: Multimethod Modeling with AnyLogic 6; AnyLogic. New York, USA: Wiley Online Library. ISBN 0-9895731-7-6. 12:248–279. doi: 10.1002/9781118762745.ch12

[CIT0010] Borshchev, A., Y. Karpov, and V. Kharitonov. 2002. Distributed simulation of hybrid systems with AnyLogic and HLA. Future Gener. Comput. Syst. 18:829–839. doi: 10.1016/s0167-739x(02)00055-9

[CIT0011] Bradhurst, R. A., S. E. Roche, I. J. East, P. Kwan, and M. G. Garner. 2016. Improving the computational efficiency of an agent-based spatiotemporal model of livestock disease spread and control. Environ. Model. Softw. 77:1–12. doi: 10.1016/j.envsoft.2015.11.015

[CIT0012] Diez Roux, A. V. 2011. Complex systems thinking and current impasses in health disparities research. Am. J. Public Health 101:1627–1634. doi: 10.2105/AJPH.2011.300149 21778505 PMC3154209

[CIT0013] Dressler, G., J. Groeneveld, C. M. Buchmann, C. Guo, N. Hase, J. Thober, and B. Müller. 2019. Implications of behavioral change for the resilience of pastoral systems—Lessons from an agent-based model. Ecol. Complexity 40:100710. doi:10.1016/j.ecocom.2018.06.002.

[CIT0014] Dumont, B., and D. R. Hill. 2001. Multi-agent simulation of group foraging in sheep: effects of spatial memory, conspecific attraction, and plot size. Ecol. Model. 141:201–215. doi: 10.1016/s0304-3800(01)00274-5

[CIT0015] Fernandez-Mena, H., G. K. MacDonald, S. Pellerin, and T. Nesme. 2020. Co-benefits and trade-offs from agro-food system redesign for circularity: a case study with the FAN agent-based model. Front. Sustain. Food Syst. 4:41. doi:10.3389/fsufs.2020.00041.

[CIT0016] Fust, P., and E. Schlecht. 2018. Integrating spatio-temporal variation in resource availability and herbivore movements into rangeland management: RaMDry—An agent-based model on livestock feeding ecology in a dynamic, heterogeneous, semi-arid environment. Ecol. Model. 369:13–41. doi: 10.1016/j.ecolmodel.2017.10.017

[CIT0017] Garcia, R., J. Aguilar, M. Toro, A. Pinto, and P. Rodriguez. 2020. A systematic literature review on the use of machine learning in precision livestock farming. Comput. Electron. Agric. 179:105826. doi:10.1016/j.compag.2020.105826.

[CIT0018] Gilbert, N., and S. Bankes. 2002. Platforms and methods for agent-based modeling. Proc. Natl. Acad. Sci. U.S.A. 99:7197–7198. doi: 10.1073/pnas.072079499 12011398 PMC128584

[CIT0019] Gross, J. E., R. R. J. McAllister, N. Abel, D. S. Smith, and Y. Maru. 2006. Australian rangelands as complex adaptive systems: a conceptual model and preliminary results. Environ. Model. Softw. 21:1264–1272. doi: 10.1016/j.envsoft.2005.04.024

[CIT0020] Groves, J. T., T. J. Goldsmith, and J. M. Carlson. 2022. How forces of a complex adaptive system affect ability to control bovine respiratory disease in feeder cattle. Vet. Clin. North Am. Food Anim. Pract. 38:295–316. doi:10.1016/j.cvfa.2022.02.006 35691630

[CIT0021] Gutiérrez, N. T., S. Rege, A. Marvuglia, and E. Benetto. 2017. Sustainable farming behaviors: an agent-based modelling and LCA perspective. In: Agent-based modeling of sustainable behaviors pp. 187–206. Switzerland: Springer International Publishing, pp. 187–206. doi:10.1007/978-3-319-46331-5_9

[CIT0022] Holland, J. H. 1992. Complex adaptive systems. Daedalus 121:17–30. doi: http://www.jstor.org/stable/20025416.

[CIT0023] Huber, R., M. Bakker, A. Balmann, T. Berger, M. Bithell, C. Brown, G. Mack, R. Seidl, C. Troost, and R. Finger. 2018. Representation of decision-making in European agricultural agent-based models. Agric. Sys. 167:143–160. 10.1016/j.agsy.2018.09.007.

[CIT0024] Jacobs, M., A. Remus, C. Gaillard, H. M. Menendez III, L. O. Tedeschi, S. Neethirajan, and J. L. Ellis. 2022. Mathematical modeling in animal nutrition: limitations and potential next steps for modeling and modelers in the animal sciences. J. Anim. Sci. 100:skac132. doi:10.1093/jas/skac132. 35419602 PMC9171330

[CIT0025] Janssen, M. A., L. N. Alessa, M. Barton, S. Bergin, and A. Lee. 2008. Towards a community framework for agent-based modelling. J. Arti. Societ. Soc. Simul. 11:6. doi:https://www.jasss.org/11/2/6.html.

[CIT0026] Kagho, G. O., M. Balac, and K. W. Axhausen. 2020. Agent-based models in transport planning: current state, issues, and expectations. Procedia Comput. Sci. 170:726–732. doi: 10.1016/j.procs.2020.03.164

[CIT0027] Kaniyamattam, K., J. Block, P. J. Hansen, and A. De Vries. 2018. Economic and genetic performance of various combinations of in vitro-produced embryo transfers and artificial insemination in a dairy herd. J. Dairy Sci. 101:1540–1553. doi: 10.3168/jds.2017-13475 29153526

[CIT0028] Lange, M., and H. H. Thulke. 2017. Elucidating transmission ­parameters of African swine fever through wild boar carcasses by combining spatio-temporal notification data and agent-based modelling. Stoch. Environ. Res. Risk Assess. 31:379–391. doi: 10.1007/s00477-016-1358-8

[CIT0029] Luke, S., C. Cioffi-Revilla, L. Panait, K. Sullivan, and G. Balan. 2005. Mason: a multiagent simulation environment. Simulation 81:517–527. doi: 10.1177/0037549705058073

[CIT0030] Lytinen, S. L., and S. F. Railsback. 2012. The evolution of agent-based simulation platforms: a review of NetLogo 5.0 and ReLogo. Paper presented at the European meeting on cybernetics and systems research. Vienna, Austria.

[CIT0031] Macal, C. M., and M. J. North. 2005. Tutorial on agent-based modeling and simulation. Paper presented at the proceedings of the winter simulation conference. Orlando, Florida, pp*-*14. doi: 10.1109/WSC.2005.1574234

[CIT0032] Marvuglia, A., A. Bayram, P. Baustert, T. N. Gutiérrez, and E. Igos. 2022. Agent-based modelling to simulate farmers’ sustainable decisions: farmers’ interaction and resulting green consciousness evolution. J. Clean Prod. 332:129847. doi: 10.1016/j.jclepro.2021.129847

[CIT0033] Masad, D., and J. Kazil. 2015. Mesa: an agent-based modeling framework. Proceeding of the 14th Python in science conference. Austin, Texas. doi: 10.1007/978-3-030-61255-9_30.

[CIT0034] Metcalf, S. S. 2007. Simulating the social dynamics of spatial disparity through neighborhood network evolution. PhD Dissertation. University of Illinois at Urbana-Champaign, Champaign, IL.

[CIT0035] Minar, N., Burkhart, R., Langton, C., and M. Askenazi. 1996. The swarm simulation system: a toolkit for building multi-agent simulations. SantaFe Institute report 06, 042.

[CIT0036] Müller-Hansen, F., J. Heitzig, J. F. Donges, M. F. Cardoso, E. L. Dalla-Nora, P. Andrade, and K. Thonicke. 2019. Can intensification of cattle ranching reduce deforestation in the amazon? insights from an agent-based social-ecological model. Ecolog. Econ. 159:198–211. doi:10.1016/j.ecolecon.2018.12.025.

[CIT0037] Namany, S., R. Govindan, L. Alfagih, G. McKay, and T. Al-Ansari. 2020. Sustainable food security decision-making: an agent-based modelling approach. J. Clean Prod. 255:120296. doi: 10.1016/j.jclepro.2020.120296

[CIT0038] Nesheim M.C. , M. Oria, and P. T. Yih, editors, 2015. Committee on a Framework for Assessing the Health, Environmental, and Social Effects of the Food System; Food and Nutrition Board; Board on Agriculture and Natural Resources; Institute of Medicine; National Research Council. A Framework for Assessing Effects of the Food System. National Academies Press. Washington D. C. PMID: 26203480. 26203480

[CIT0039] Noe, E., and H. F. Alrøe. 2003. Farm enterprises as self-organizing systems: a new transdisciplinary framework for studying farm enterprises? Int. J. Sociol. Agric. Food 11:3–14. doi:10.48416/ijsaf.v11i.325.

[CIT0040] North, M. J., Howe, T. R., Collier, N. T., and J. R. Vos. 2005. The repast simphony development environment. Paper presented at the Proceedings of the Agent 2005 Conference on Generative Social Processes, Models, and Mechanisms, Argonne, Illinois. ANL/DIS-06-5, ISBN 0-9679168-6-0.

[CIT0041] North, M. J, Tatara, E., Collier, N. T., and J. Ozik. 2007. Visual agent-based model Development with Repast Simphony, ANL/DIS/CP-60520, Argonne National Lab, Argonne, IL.

[CIT0042] North, M. J., N. T. Collier, J. Ozik, E. R. Tatara, C. M. Macal, M. Bragen, and P. Sydelko. 2013. Complex adaptive systems modeling with repast simphony. Complex Adap. Syst. Model. 1:1–26. doi:10.1186/2194-3206-1-3.

[CIT0043] Ojeda-Rojas, O. A., A. M. Gonella-Diaza, D. Bustos-Coral, G. L. Sartorello, T. S. Reijers, G. Pugliesi, and A. H. Gameiro. 2021a. An agent-based simulation model to compare different reproductive strategies in cow-calf operations: technical performance. Theriogenology 160:102–115. doi:10.1016/j.theriogenology.2020.10.035. 33212420

[CIT0044] Ojeda-Rojas, O. A., A. M. Gonella-Diaza, D. Bustos-Coral, G. L. Sartorello, T. S. Reijers, G. Pugliesi, and A. H. Gameiro. 2022. An agent-based simulation model to compare different reproductive strategies in cow-calf operations: economic performance. Theriogenology 189:11–19. doi:10.1016/j.theriogenology.2022.06.002. 35738033

[CIT0045] Ormerod, P., and B. Rosewell. 2009. Validation and verification of agent-based models in the social sciences. Paper presented at the Epistemological Aspects of Computer Simulation in the Social Sciences: Second International Workshop, EPOS 2006, Brescia, Italy, October 5-6, 2006, Revised Selected and Invited Papers. Brescia, Italy, 130–140.

[CIT0046] Osorio-Doblado, A. M., K. P. Feldmann, J. M. Lourenco, R. L. Stewart, W. B. Smith, L. O. Tedeschi, F. L. Fluharty, and T. R. Callaway. 2023. Forages and Pastures Symposium: Forage biodegradation: advances in ruminal microbial ecology. J. Anim. Sci. 101. skad178. doi: 10.1093/jas/skad178 37257501 PMC10313095

[CIT0047] Roche, S. E., M. G. Garner, R. M. Wicks, I. J. East, and K. de Witte. 2014. How do resources influence control measures during a simulated outbreak of foot and mouth disease in Australia? Prev. Vet. Med. 113:436–446. doi: 10.1016/j.prevetmed.2013.12.003 24412502

[CIT0048] Schelling, T. C. 1971. Dynamic models of segregation. J. Math. Sociol. 1:143–186. doi: 10.1080/0022250x.1971.9989794

[CIT0049] Schreinemachers, P., and T. Berger. 2011. An agent-based simulation model of human–environment interactions in agricultural systems. Environ. Model. Softw. 26:845–859. doi: 10.1016/j.envsoft.2011.02.004

[CIT0050] Soriano, B., A. Garrido, D. Bertolozzi-Caredio, F. Accatino, F. Antonioli, V. Krupin, P. M. Mranda, J. Rommel, A. Spiegel, M. Tudor, et al. 2023. Actors and their roles for improving resilience of farming systems in europe. J. Rural Stud. 98:134–146. doi:10.1016/j.jrurstud.2023.02.003.

[CIT0051] Sun, Z., I. Lorscheid, J. D. Millington, S. Lauf, N. R. Magliocca, J. Groeneveld, J. Schulze, J. Schulze, and C. M. Buchmann. 2016. Simple or complicated agent-based models? A complicated issue. Environ. Model. Softw. 86:56–67. doi:10.1016/j.envsoft.2016.09.006.

[CIT0052] Tedeschi, L. O. 2006. Assessment of the adequacy of mathematical models. Agric. Sys. 89:225–247. doi: 10.1016/j.agsy.2005.11.004

[CIT0053] Tedeschi, L. O. 2019. ASN-ASAS SYMPOSIUM: FUTURE OF DATA ANALYTICS IN NUTRITION: Mathematical modeling in ruminant nutrition: approaches and paradigms, extant models, and thoughts for upcoming predictive analytics. J. Anim. Sci. 97:1921–1944. doi: 10.1093/jas/skz092 30882142 PMC6488328

[CIT0054] Tedeschi, L. O. 2023. Review: the prevailing mathematical modelling classifications and paradigms to support the advancement of sustainable animal production. Animal 100813. doi: 10.1016/j.animal.2023.100813 37169649

[CIT0055] Tedeschi, L. O., and D. G. Fox. 2020. The ruminant nutrition system: Volume I - An applied model for predicting nutrient requirements and feed utilization in ruminants. (3rd ed.). Ann Arbor, MI, USA: XanEdu.

[CIT0056] Tedeschi, L. O., P. L. Greenwood, and I. Halachmi. 2021. Advancements in sensor technology and decision support intelligent tools to assist smart livestock farming. J. Anim. Sci. 99. skab038. doi: 10.1093/jas/skab038 33550395 PMC7896629

[CIT0057] Ten Broeke, G., G. Van Voorn, and A. Ligtenberg. 2016. Which sensitivity analysis method should I use for my agent-based model? J. Arti. Societ. Soc. Simul. 19:5. doi:10.18564/jasss.2857.

[CIT0058] Thiele, J. C., W. Kurth, and V. Grimm. 2014. Facilitating parameter estimation and sensitivity analysis of agent-based models: a cookbook using NetLogo and R. J. Arti. Societ. Soc. Simul. 17:11. doi:10.18564/jasss.2503.

[CIT0059] Thompson, M. 2021. Agent based modeling to address emerging threats from antimicrobial resistance to the sustainability of the beef industry. Thesis submitted to University of Saskatchewan, Saskatoon.

[CIT0060] Wilensky, U. 1999. NetLogo. The center for connected learning and computer-based modeling. Evaston, Illinois, Northwestern University https://ccl.northwestern.edu/#:~:text=The%20CCL%20is%20a%20research,here%20and%20at%20other%20universities.

[CIT0061] Wilensky, U., and W. Rand. 2007. Making models match: replicating an agent-based model. J. Arti. Societ. Soc. Simul. 10:2. doi:https://www.jasss.org/10/4/2.html. 10.18564/jasss.4352 PMC7668565 33204215

[CIT0062] Wilensky, U., and W. Rand. 2015. An introduction to agent-based modeling: Modeling natural, social, and engineered complex systems with NetLogo. Cambridge, Massachusetts: Mit Press. http://www.jstor.org/stable/j.ctt17kk851.

[CIT0063] Wiltshire, S. W. 2018. Using an agent-based model to evaluate the effect of producer specialization on the epidemiological resilience of livestock production networks. PLoS One 13:e0194013. doi: 10.1371/journal.pone.0194013 29522574 PMC5844541

[CIT0064] Windrum, P., G. Fagiolo, and A. Moneta. 2007. Empirical validation of agent-based models: alternatives and prospects. J. Art. Societies Soc. Simul. 10:8. doi:https://www.jasss.org/10/2/8.html.

[CIT0065] Yang, Q., D. Gruenbacher, J. L. H. Stamm, G. L. Brase, S. A. DeLoach, D. E. Amrine, and C. Scoglio. 2019. Developing an agent-based model to simulate the beef cattle production and transportation in southwest Kansas. Physica A 526:120856. doi:10.1016/j.physa.2019.04.092.

[CIT0066] Yang, S., L. Yu, G. Leng, and H. Qiu. 2021. Livestock farmers’ perception and adaptation to climate change: panel evidence from pastoral areas in China. Clim. Change 164:1–24. doi:10.1007/s10584-021-02992-7.34334847

[CIT0067] Yu, R., A. J. Evans, and N. Malleson. 2019. An agent-based model for assessing grazing strategies and institutional arrangements in Zeku, China. Agric. Sys. 171:135–142. doi:10.1016/j.agsy.2019.02.004.

[CIT0068] Zhang, W., A. Valencia, and N. Chang. 2021. Synergistic integration between machine learning and agent-based modeling: a multidisciplinary review. IEEE Trans. Neural Networks Learn. Syst. 34:2170–2190. doi:10.1109/tnnls.2021.3106777. 34473633

