# Supplementary material for: ASAS-NANP symposium: mathematical modeling in animal nutrition: agent-based modeling for livestock systems: the mechanics of development and application
Source: J Anim Sci. 2023 Nov 22;101:skad321. doi: 10.1093/jas/skad321 (PMC10664392; doi:10.1093/jas/skad321)
Supplement: skad321_suppl_Supplementary_Appendixs_1 [file skad321_suppl_supplementary_appendixs_1.docx]

**Appendix.** The Net Logo code used to develop the agent-based model of rumen fermentation is given below. The predator agents in the model are non-fiber bacteria (feeding on starch), protozoa, and bacteria which feeds on fiber. Predator agents feed on substrates (starch, fiber, and two different types of bacteria) and influences the emergent behavior observed after simulation (Figure 2).

breed [non-fiber-bacteria a-non-fiber-bacteria]

breed [protozoa a-protozoa]

breed [fiber-bacteria a-fiber-bacteria]

turtles-own [energy]

patches-own [fiber-patch? grain-patch? fiber-amount grain-amount]

to setup

clear-all

setup-patches

create-non-fiber-bacteria number-of-non-fiber-bacteria [

setxy random-xcor random-ycor

set color yellow

set shape “circle”

set energy 50

]

create-protozoa number-of-protozoa [

setxy random-xcor random-ycor

set color white

set shape “triangle”

set energy 100

]

create-fiber-bacteria number-of-fiber-bacteria [

setxy random-xcor random-ycor

set color blue

set shape “circle”

set energy 50

]

reset-ticks

end

to setup-patches

ask patches [

ifelse random-float 1 < 0.5 [

set fiber-patch? true

set grain-patch? false

set pcolor green

] [

set fiber-patch? false

set grain-patch? true

set pcolor orange

]

set fiber-amount 100

set grain-amount 100

]

end

to go

if not any? turtles [

stop

]

ask protozoa [

move

check-if-dead

eat-bacteria

reproduce

]

ask non-fiber-bacteria [

move

check-if-dead

eat-grain-patch

reproduce

]

ask fiber-bacteria [

move

check-if-dead

eat-fiber-patch

reproduce

]

regrow-fiber-patch

regrow-grain-patch

tick

my-update-plots ;; plot the population counts

end

to move

rt random 90

lt random 90

forward 1

set energy energy - 1

end

to check-if-dead

if energy <= 0 [

die

]

end

to recolor-fiber-patch

ask patches with [fiber-patch?] [

set pcolor green

]

end

to regrow-fiber-patch

ask patches with [not fiber-patch?] [

if random-float 1 < fiber-patch-regrowth-rate [

set fiber-patch? true

set pcolor green

]

]

end

to recolor-grain-patch

ask patches with [grain-patch?] [

set pcolor orange

]

end

to regrow-grain-patch

ask patches with [not grain-patch?] [

if random-float 1 < grain-patch-regrowth-rate [

set grain-patch? true

set pcolor orange

]

]

end

to eat-bacteria

let non-fiber-bacteria-here turtles with [breed = non-fiber-bacteria]

let fiber-bacteria-here turtles with [breed = fiber-bacteria]

let total-affinity 0.6 * count non-fiber-bacteria-here + 0.4 * count fiber-bacteria-here

if total-affinity > 0 [

let target-bacteria nobody

if random-float 1 < 0.6 [

set target-bacteria one-of non-fiber-bacteria-here

] if random-float 1 >= 0.6 [

set target-bacteria one-of fiber-bacteria-here

]

if target-bacteria != nobody [

ask target-bacteria [

die

]

set energy energy + energy-gain-from-bacteria

]

]

end

to eat-fiber-patch

ifelse any? patches with [pxcor = [pxcor] of myself and pycor = [pycor] of myself and fiber-patch?] [

let target one-of patches with [pxcor = [pxcor] of myself and pycor = [pycor] of myself and fiber-patch?]

set energy energy + energy-gain-from-fiber-patch

ask target [

set fiber-patch? false

;; decrement the fiber amount

set fiber-amount fiber-amount - energy-gain-from-fiber-patch

recolor-fiber-patch

]

] [

set energy energy - 1

]

end

to eat-grain-patch

ifelse any? patches with [pxcor = [pxcor] of myself and pycor = [pycor] of myself and grain-patch?] [

let target one-of patches with [pxcor = [pxcor] of myself and pycor = [pycor] of myself and grain-patch?]

set energy energy + energy-gain-from-grain-patch

ask target [

set grain-patch? false

;; decrement the grain amount

set grain-amount grain-amount - energy-gain-from-grain-patch

recolor-grain-patch

]

] [

set energy energy - 1

]

end

to reproduce

if energy >= 100 [

hatch 1 [

set color [color] of myself

set energy 50

rt random 180

fd 1

]

set energy energy - 50

]

end

to my-update-plots

set-current-plot-pen "non-fiber-bacteria"

plot count non-fiber-bacteria

set-current-plot-pen “fiber-bacteria”

plot count fiber-bacteria

set-current-plot-pen “protozoa”

plot count protozoa

set-current-plot-pen “grain-patch”

plot sum [grain-amount] of patches / (count patches with [grain-patch?]) ;; scaling factor so plot looks nice

set-current-plot-pen “fiber-patch”

plot sum [fiber-amount] of patches / (count patches with [fiber-patch?]) ;; scaling factor so plot looks nice

end
